# Integration site analysis in Japanese HTLV-1 infected asymptomatic carriers and HAM/TSP patients

**DOI:** 10.1186/1742-4690-11-S1-P69

**Published:** 2014-01-07

**Authors:** Heather Niederer, Daniel Laydon, Anat Melamed, Marjet Elemans, Becca Asquith, Masao Matsuoka, Charles Bangham

**Affiliations:** 1 Department of Medicine, Imperial College London, London, UK; 2 Institute for Viral Research, Kyoto University, Kyoto, Japan

## 

The proviral load (PVL) in HTLV-1 infection is strongly correlated with odds of the inflammatory disease HAM/TSP. Individuals with HLA class I alleles which can bind HBZ have reduced PVL and reduced risk of HAM/TSP. We tested the hypothesis that a strong CD8^+^ T-cell response to HBZ alters the frequency distribution of infected T-cell clones and selects the genomic environment of the proviral integration site (IS) in vivo. We used our recently described high-throughput protocol to map and quantify IS in 95 HAM/TSP patients and 68 asymptomatic carriers (ACs) from Kagoshima, Japan, and 75 ACs from Kumamoto, Japan. Individuals with 2 or more HLA class I alleles predicted to bind HBZ were classified as ‘strong’ HBZ binders. The results suggest that the predicted strength of HBZ binding does not influence the overall clone frequency distribution. However, clonal abundance was correlated with frequency of proviral integration within transcriptionally active areas in weak HBZ binders, but not strong HBZ binders. IS in transcriptionally active areas were more frequent in ACs than HAM/TSP, irrespective of clone abundance or HBZ binding strength, and were more frequent in strong HBZ binders than weak binders in low-abundance clones only. We propose that proviral integration in transcriptionally active areas of the genome conveys a proliferation advantage on the infected T-cell clone, but the equilibrium abundance of that clone is limited by stronger immune selection in ACs. A strong CD8^+^ T-cell response to HBZ may remove this advantage, perhaps by selecting against high HBZ expression.

